# Varied Response of EEG Rhythm to Different tDCS Protocols and Lesion Hemispheres in Stroke Subjects with Upper Limb Dysfunction

**DOI:** 10.1155/2022/7790730

**Published:** 2022-07-30

**Authors:** Chunfang Wang, Yuanyuan Chen, Peiqing Song, Hongli Yu, Jingang Du, Ying Zhang, Changcheng Sun

**Affiliations:** ^1^Rehabilitation Medical Department, Tianjin Union Medical Centre; Rehabilitation Medical Research Center of Tianjin, Tianjin, China; ^2^Tianjin International Joint Research Center for Neural Engineering, Academy of Medical Engineering and Translational Medicine, Tianjin University, Tianjin, China; ^3^State Key Laboratory of Reliability and Intelligence of Electrical Equipment, Hebei University of Technology, Tianjin, China

## Abstract

Transcranial direct current stimulation (tDCS) provides a way to modulate the cortical activity and promote motor rehabilitation following stroke. However, evidence indicates that the response to tDCS is highly variable. This study was aimed at exploring rhythmic response of Electroencephalography (EEG) to three tDCS protocols in stroke subjects. We hypothesize that tDCS protocols may interact with stoke characteristics, and electrode placement may affect cortical activity which could be reflected by the EEG rhythm. 32 subjects with unilateral stroke were recruited to a single-blinded, randomized, and controlled crossover experiment. All of the subjects underwent four tDCS protocols (anodal (atDCS), cathodal (ctDCS), and bilateral tDCS (bi-tDCS) and sham) with an interval of at least 1 week. Resting-state EEG was acquired before and after the stimulation. We tested the change of EEG spectral power after tDCS and the difference of change among four protocols using the paired-sample *t*-test and repeated measures analysis of variance. Then, we investigated the clinical factors affecting the above changes using the linear and quadratic regression model. According to the results, EEG responded to atDCS and bi-tDCS protocols on alpha and beta rhythm and subjects with a left lesion had higher response than those with the right lesion. Besides that, the change of alpha and beta power after atDCS and of beta power after bi-tDCS showed association with clinical characteristics only in subjects with the left lesion. In conclusion, the study found varied EEG response with different protocols, lesion hemispheres, and other clinical characteristics supporting the individualized cortical oscillatory effect induced by tDCS.

## 1. Introduction

Hemiplegia occurs frequently after stroke, and the recovery of upper limb motor function becomes the most issue addressed in neurorehabilitation. Among the recovery treatment measures, transcranial direct current stimulation (tDCS) provides a new way for the modulation of brain activity and promotion of motor rehabilitation [1–3]. However, its aftereffects of plasticity change on stroke subjects are not well understood, and the protocols treating for motor rehabilitation are not yet standardized.

According to the interhemispheric inhibition (IHI) poststroke, tDCS promotes neuroplasticity poststroke through either increasing ipsilesional excitability or decreasing contralesional excitability or both at the same time via bihemispheric tDCS [3]. Thus, three different electrode placement protocols were proposed accordingly: first, the anodal electrode is placed over the primary motor cortex (M1) region of the cerebral hemisphere of the affected side, upregulating the neuronal excitability of the affected hemisphere, and the cathode is set as a reference electrode. Second, the cathode electrode is placed over the M1 region of the brain hemisphere of the healthy side, downregulating the neuronal excitability of the healthy side, and the anode is set as a reference electrode. Third, the anode is placed over the affected side, and the cathode is placed on the healthy side, balancing the neuronal excitability of two hemispheres.

Although the above protocols are used a lot in clinical rehabilitation for poststroke subjects with hemiplegia, the treatment results of tDCS are highly variable across individuals. Some review literature showed positive response with long-term beneficial effects on motor function recovery [4], but some did not [1, 3]. Others suggest that heterogeneity in the clinical profile of the population may be an important reason for different results in terms of the tDCS efficacy, and some tDCS protocols may fit for specific stroke individuals [5, 6]. Much effort has therefore been dedicated to explore the related factors affecting tDCS efficacy. Polar of stimulation electrodes, lesion hemisphere, time since stroke, and the level of motor impairment were supposed to be possible reasons causing interindividual variability of tDCS effects [7–9], but the interaction between these clinical factors and tDCS efficacy is not well clarified.

Understanding cortical activity induced by tDCS could help to answer the above question, and it is possible to measure cortical activity after tDCS based on brain imaging techniques such as Functional Magnetic Resonance Imaging (fMRI) and Electroencephalography (EEG). Recently, there has been increasing interest in exploring the local and global modulation effects of tDCS on neural plasticity using fMRI or EEG [1, 10]. Increased functional activity in motor cortex areas and interhemispheric connectivity were found after tDCS in stroke subjects using fMRI [11, 12]. For EEG studies, researchers mainly focused on tDCS effects in healthy subjects and found oscillation changes in theta, alpha, and beta bands over rest and task states [13–15]. Stronger connectivity of ipsilesional motor and the parietal cortex and contralesional frontotemporal cortex was found to be associated with increase in corticospinal excitability following anodal tDCS in chronic stroke survivors [16].

In this study, we aimed to explore the modulation effect of tDCS to unilateral stroke subjects with upper limb motor dysfunction. The alpha (8-13 Hz) and beta rhythms (13-30 Hz) are associated with function of the motor system, and their decrease was found to be related to motor deficits in poststroke subjects [17–19]. Previous studies find that different electrode placement protocols induced alpha and beta rhythms differently in healthy subjects [20, 21], and different protocols showed varied rehabilitation results to stroke subjects. Therefore, we hypothesize that tDCS could improve brain activity by modulating the alpha and beta rhythms and try to find the interaction between stroke characteristics and electrode placement protocols through cortical electrical activity. Thus, we investigated the EEG response in frequency bands after three kinds of tDCS protocols and examined if stroke characteristics like lesion hemisphere, poststroke time, and severity of upper limb impairment are factors of heterogeneity to tDCS effects and if EEG could be a tool to evaluate tDCS effects on cortical response. We acquired resting-state EEG before and after three kinds of tDCS protocols (anodal, cathodal, and bilateral) and a sham condition. Then, we tested and compared the spectral power change of EEG rhythms induced by different tDCS protocols separately for left- and right-affected subjects. Finally, we analyzed the clinical factors affecting the EEG response of tDCS.

## 2. Methods

### 2.1. Subjects

Subjects with ischemic stroke were recruited from the Rehabilitation and Neurology Department of Tianjin Union Medical Centre according to the following criteria: (1) first-ever diagnosed with unilateral subcortical ischemic stroke according to MRI, (2) aged >18 and <75 years, (3) subjects in the chronic stage (>3 months poststroke), and (4) no surgical treatment. The exclusion criteria were as follows: (1) with metal implants in the skull; (2) ruptured scalp skin; (3) taking antidepressant, antianxiety, and other drugs affecting the nervous system; and (4) subjects with mental disorders or unable to cooperate. All subjects underwent upper limb impairment assessment using upper extremity Fugl-Meyer (FM) assessment of upper extremity and activity of daily living assessment using the modified Barthel Index (MBI). All subjects were informed of all aspects of the experiment including the possibility of minor adverse effects related to tDCS, such as transient sensations of itching, burning, and prickling on the scalp. The study was approved by the ethics committee of Nankai University (the ethical approval number is NKUIRB2018016). All subjects signed a written informed consent form before the experiment started. Subjects finishing at least three sessions of the experiment were taken on board, and 32 subjects with 16 left- and 16 right-hemispheric lesions were included in the study. Among them, there was one subject who underwent three sessions of the experiment lacking the atDCS session with personal reason, and the others completed all the experimental sessions.

### 2.2. Transcranial Direct Current Stimulation Protocol

We designed a single-blinded, randomized, and controlled crossover experiment consisting of four within-subject experimental sessions: three active protocols (anodal, atDCS; cathodal, ctDCS; and bilateral, bi-tDCS) and a sham condition. The sham stimulation served as a control to isolate the effects of current stimulation from the placebo and somatosensory effects that could arise from tDCS application. We generated a random table using a block random method through the MATLAB program to determine the implementation order of atDCS, ctDCS, bi-tDCS, and sham conditions. Supplemental Table 1 shows the randomized stimulation sequence of four sessions of the experiment. All subjects underwent the above four sessions in the order shown in the randomized table and were blinded to the condition. The interval between each of the four conditions was at least 1 week. The placement protocols of the four conditions were the same as one of our previous studies [22]: (1) atDCS: the anode electrode was placed over the primary motor cortex (M1) of the ipsilesional side, and the cathode electrode was placed over the lateral supraorbital as the reference electrode; (2) ctDCS: the cathode was placed over the M1 of the contralateral hemisphere, and the anode was placed over the lateral supraorbital as the reference electrode; ③ bi-tDCS: the anode was placed over the M1 of the ipsilesional side, and the cathode was placed over the M1 of the contralateral hemisphere; and (4) sham: this is consistent with the placement of atDCS. The M1 of the left hemisphere was set as the C3 according to the international standard 10-20 EEG system, and the M1 of the right hemisphere was set as the location of C4.

Direct current was transferred by a saline-soaked pair of surface sponge electrodes (5 cm × 7 cm) and delivered by a specially developed battery-driven constant-current electrical stimulator (neuroConn, Germany). The stimulation parameter was 1.75 mA (current density 0.5 A/m^2^) over 20 min. As the sham condition, the electrodes were located in the same positions as in the atDCS, but the current was supplied only for the first 46 s (8 s ramp up, 30 s of DC stimulation, and 8 s ramp down) to make subjects feel a tingling sensation at the beginning of the stimulation [13].

### 2.3. EEG Acquisition and Processing

Resting-state EEG with eyes open was acquired for 5 minutes before and after tDCS using the Neuroscan EEG system (made by US). Sixty-two electrodes were placed on the scalp according to the International 10-20 position system. A pair of vertical electrooculogram (VEOG) and a pair of horizontal electrooculogram (HEOG) electrodes were also recorded to remove ocular artifacts in a later processing step for artifact removal. The electrode impedance was kept below 10 k*Ω*. The EEG signal was amplified with a band pass of 0.1-70 Hz and sampled at 1000 Hz. The forehead was set as the ground, and linked earlobes were set as reference electrodes.

Spectral power of delta, theta, alpha1, alpha2, beta1, and beta2 band was calculated based on Fast Fourier Transform (FFT) to analyze statistically. Preprocessing was conducted first following the steps as follows: (1) desample EEG data to 250 Hz, (2) extract the data between 0.25 Hz and 45 Hz using a finite impulse response (FIR) filter, and (3) use independent component analysis (ICA) to remove artifacts including ophthalmic and EMG interference. Then, digital FFT-based power spectrum analysis (Welch technique, Hamming windowing function) was conducted to compute power spectrum density (PSD) average value of each EEG channel separately with NFFT = 1024, window = 256, and 50% overlap. After that, spectral power of delta (1-4 Hz), theta (4-8 Hz), alpha1 (8-10 Hz), alpha2 (10-13 Hz), beta1 (13-20 Hz), and beta2 (20-30 Hz) rhythms was calculated according to the frequency bands. More details have been described in our previous study [22]. All of the above processes were conducted through the MATLAB software and EEGLAB tool box.

### 2.4. Statistical Analysis

The comparison of the proportions of the lesion site, gender, and handedness was performed using the chi-square test, and that of the clinical characteristics of time poststroke, upper extremity Fugl-Meyer, and other assessment scores between subjects with left and right hemispheric lesions was made by independent sample *t*-tests. According to the Kolmogorov-Smirnov test, data used for the *t*-test was normally distributed.

The paired-sample *t*-test was used to compare the spectral power of each frequency band before and after tDCS. The four tDCS protocols and two hemispheric lesion groups were conducted, respectively. According to the Kolmogorov-Smirnov test, data of spectral power was normally distributed. The Benjamini and Hochberg method of False Discovery Rates (BHFDR) was used to correct the multiple comparisons.

Repeated measures analysis of variance (ANOVA) with between-subjects factors of the lesion hemisphere (left and right) and within-subjects factors of tDCS protocols (atDCS, ctDCS, bi-tDCS, and sham) was used to test the spectral power difference among tDCS protocols and lesions and the interaction between the lesion hemisphere and tDCS protocol. Before the ANOVAs, Mauchly's test of sphericity was used to test the covariance matrix sphericity. If the spherical assumption was not satisfied, the Greenhouse-Geisser (G-G) method was used to adjust the degree of freedom (DF) to reduce the probability of type I error.

Linear and quadratic regression models were used and compared to test the relation between the change of spectral power and clinical factors of poststroke time, FM, and MBI scores. Age and gender were used as covariables. Groups of the left lesion and right lesion were subjected to the above analysis, respectively.

All of the above statistical analyses were performed using SPSS software.

## 3. Results

### 3.1. Basic Information and Descriptive Data

All subjects tolerated the stimulation well, not reporting any 5discomfort during the experiment. Their demographic and clinical characteristics are shown in Table 1. There was no significant difference between the two groups of the left hemispheric lesion and right hemispheric lesion in those characteristics (*p* > 0.05).

### 3.2. EEG Response to Different tDCS Protocols

**Figure 1:**
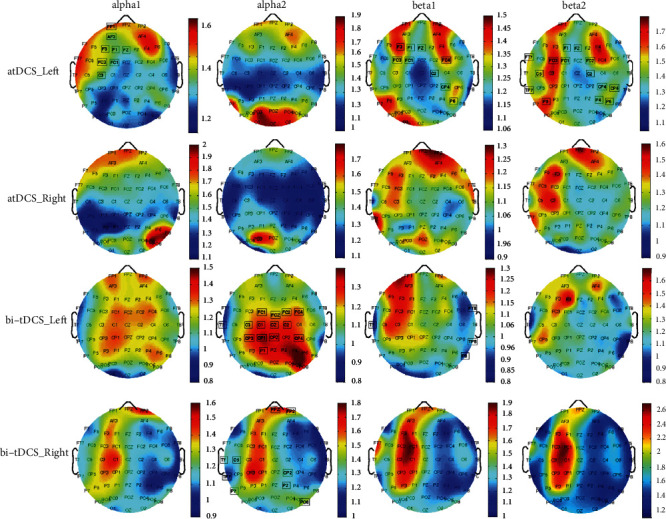
Cortical activity change of alpha (alpha1 (8-10 Hz) and alpha2 (10-13 Hz)) and beta (beta1 (13-20 Hz) and beta2 (20-30 Hz)) bands induced by anodal transcranial direct current stimulation (atDCS) and bilateral transcranial direct current stimulation (bi-tDCS) in stroke subjects with the left hemispheric lesion and right hemispheric lesion. Color of the brain topographic map represents the spectral power ratio of post- to prestimulation. The bordered labels represent regions in which spectral power was significantly different between post- and prestimulation (*p* < 0.05).

According to the results, subjects with the left hemispheric lesion had response to atDCS and bi-tDCS and those with the right hemispheric lesion have response to bi-tDCS. For subjects with the left lesion, the power of alpha1 in frontal and frontal-central regions of the left hemisphere and the power of beta1 and beta 2 in frontal, frontal-central, central, and partial regions of the left hemisphere increased significantly (*p* < 0.05) after atDCS; the power of alpha2 in frontal-central, central, and central-partial regions of the left hemisphere increased significantly after bi-tDCS. For subjects with the right lesion, the power of alpha2 in prefrontal, central, and partial regions of the right hemisphere increased significantly (*p* < 0.05) after bi-tDCS.  Figure 1 shows the result of comparison between post- and prestimulation of atDCS and bi-tDCS. None of the two groups had response to ctDCS or sham stimulation (*p* > 0.05).

### 3.3. Spectral Power Difference among tDCS Protocols and Lesions

Results of repeated measures ANOVA showed that the effect of spectral power difference was mainly focused on the alpha band among different tDCS protocols. Specifically, spectral power of the alpha 1 band in frontal-temporal areas of the left hemisphere and the frontal area of the right hemisphere was significantly different (*p* < 0.05) between atDCS and sham; spectral power of the alpha 2 band in the frontal area of the right hemisphere and the occipital area of the left hemisphere was significantly different (*p* < 0.05) between atDCS and sham. No significant difference was found between left and right hemispheric lesions.  Table 2 shows the statistic results of channels with statistical differences. Due to the limit of paper space, some results with nonsignificant differences were ignored.

### 3.4. Relation between EEG Response and Clinical Factors

**Figure 2:**
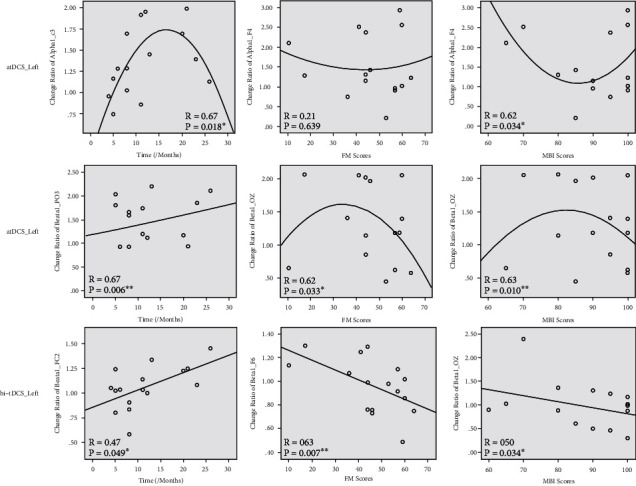
Scatter plots and fitted curves of clinical characters and the change of spectral power ratio of post- to prestimulation. atDCS_Left represents the group of stroke subjects with the left hemispheric lesion induced by anodal transcranial direct current stimulation (atDCS), and bi-tDCS_Left represents the group of stroke subjects with the left hemispheric lesion induced by bilateral transcranial direct current stimulation (bi-tDCS). Time represents the time poststroke; FM scores represent the Fugl-Meyer scores (upper extremity part); MBI scores represent the modified Barthel Index.

The relation of EEG response and clinical factors was only limited to the change of spectral power after atDCS and bi-tDCS in stroke subjects with the left hemispheric lesion.  Figure 2 shows the fitted relation between clinical characteristics of stroke subjects and the change of spectral power after the stimulation. We found that the change of alpha1 power induced by atDCS was related to poststroke time and MBI scores following the quadratic regression model (*p* < 0.05). Subjects with 10-20 months after stroke had higher alpha1 response, and MBI scores of 80-90 had lower response to atDCS. The change of beta1 power induced by atDCS was related to poststroke time following the linear regression model and FM scores and MBI scores following the quadratic regression model (*p* < 0.05). Subjects with longer time since stroke, moderate upper limb dysfunction (FM scores about 40), and daily living ability (MBI scores of 80-90) had higher beta1 response to atDCS. The change of beta1 power induced by bi-tDCS was related to poststroke time, FM scores, and MBI scores following the linear regression model (*p* < 0.05). Subjects with longer time since stroke, severe upper limb dysfunction, and daily living ability had higher beta1 response to bi-tDCS. We found no significant relation between clinical characteristics and the change of spectral power in stroke subjects with the right hemispheric lesion (*p* > 0.05).

## 4. Discussion

In this study, we hypothesize that tDCS protocols may interact with stoke characteristics and electrode placement may affect cortical activity which could be reflected by the EEG rhythm. Our results showed that EEG responded to atDCS and bi-tDCS protocols on alpha and beta rhythm and subjects with the left lesion had higher response than those with the right lesion. Besides that, the change of alpha and beta power after atDCS and of beta power after bi-tDCS showed association with clinical characteristics only in subjects with the left lesion. The change of EEG rhythm may be one important characteristic to evaluate tDCS effects on cortical response.

### 4.1. Increase in Alpha and Beta Power after tDCS in Poststroke Subjects

According to our result, the EEG response to tDCS was focused on increased alpha and beta power. The alpha band of EEG has been proven by several studies to be a brain rhythm involved in several cerebral functions, ranging from sensory-motor processing to memory formation [17, 23]. The decrease in the alpha band and beta synchrony was found to be related to motor deficits in poststroke subjects, and their increase reflected changes of sensory-motor processing [18, 19]. Connectivity in the alpha band has been proven to be associated with atDCS response strongly in stroke subjects, and high beta connectivity could predict response to tDCS and motor learning in healthy adults [24, 25]. Intracortical facilitation mediated by NMDA receptors was proven to be the underlying mechanism of direct current- (DC-) induced excitability changes, which may result in the observed aftereffects on alpha and beta bands [26]. The above mechanism may result in an improvement of synaptic efficiency, paired to an increased high frequency power, explaining the improvement in motor performance previously described after tDCS over the motor cortex [27].

### 4.2. EEG Response to Different tDCS Protocols

Studies on the electrical response induced by tDCS were mainly focused on healthy subjects. For atDCS, an increase in low alpha power was found after the stimulation [20], which is consistent with our study. Some studies supported the increase in alpha power [13, 20, 28, 29] and beta power [13] in frontal, sensorimotor, and parieto-occipital regions after the stimulation. But some found different cortical changes after tDCS, like increase in delta [21] and theta bands [29, 30]. For bi-tDCS, some studies reported an increase in alpha and theta power and decrease in beta power [31, 32]. For ctDCS, increased delta and theta power in the frontal region was reported after the stimulation [21].

Some researchers compared the electrical response of different protocols and found specific performance to each protocol. Notturno et al. [20] found an increase in low alpha band power after atDCS, but not after ctDCS, which is consistent with our result. Some studies reported that both atDCS and ctDCS could modulate cortical activity [21, 33]. Other studies compared the long-term effect of three protocols on motor recovery after stroke and found no consistent result [4, 34, 35]. Inconsistent results for the spectral power change may be due to the difference in stimulation target, like M1 [20], DLPFC [31, 32], posterior parietal [13], occipital [28], and temporoparietal junction [21] regions. Besides that, heterogeneity of subjects and protocols of stimulation time and current density were also possible causes of the confounding results, and stroke subjects may have specific response to tDCS due to the damage area.

The above factors may also lead to difference in response areas of the brain and population of the stroke after tDCS. Previous studies reported sensorimotor and parieto-occipital regions after atDCS over the left M1 region [20] and frontal region of the right hemisphere after bi-tDCS over DLPFC with the left anode and right cathode [32]. According to the study, atDCS showed a widespread modulation effect in frequency bands and related regions to left-affected stroke, and bi-tDCS showed a wide range of affected subjects in stroke of both hemispheric lesions. Specific modulation effects implied individualized protocols to stroke subjects in their upper limb recovery.

### 4.3. The Relation between EEG Response and Clinical Characteristics

Our finding indicates that clinical characteristics of poststroke time, upper limb dysfunction, and daily living ability were associated with the power change of alpha1 and beta1 bands after atDCS and bi-tDCS in stroke subjects with the left hemispheric lesion following linear or quadratic relation and may offer prediction for modulation effect of tDCS. Previous studies have found that tDCS efficacy varies with time after stroke, nature and location of stroke, and level of motor impairment [36–38] and also demonstrated the association between alpha oscillation and clinical status, motor performance, and functional recovery [18, 19, 39]. EEG measures of motor cortical connectivity were proven to be strongly related to motor deficits and their improvement with therapy after stroke [40]. As patterns of neural recovery may differ for individuals based on the severity of their stroke [41, 42], our results may help in development of an approach to identify subjects who respond well to a certain tDCS protocol. However, the potential benefit required further investigation considering that our evidence only supported the relation between electrical response and clinical status.

## 5. Conclusion and Limitation

The study found varied EEG response with different protocols, lesion hemispheres, and other clinical characteristics supporting the individualized cortical oscillatory effect induced by tDCS. However, it is not easy to interpret all the findings because of the limited references about modulation effect of spontaneous cortical activity in stroke subjects. In addition, due to the difference in the position of anodal and cathodal electrodes among the four protocols, the operator has to know the stimulation protocol before each session began, which is a limitation of the study. Besides that, the study could not yet answer the relation between varied EEG response and upper limb improvement with tDCS intervention, and we will design a longitudinal study to explore it in our next work, which might help to improve clinical decision-making.

## Figures and Tables

**Table 1:** Participant characteristics in this study.

	Left (*N* = 16)	Right (*N* = 16)	*χ* ^2^/*T*	*p* value
Site, BG/BS (%)	11/5 (68.75)	13/3 (81.25%)	0.667	0.414
Gender, M/F (%)	10/6 (62.5%)	12/4 (75%)	0.582	0.446
Handedness, R/L (%)	14/2 (87.5%)	14/2 (87.5%)	0	1
Age	56.19 ± 10.30	60.50 ± 8.7	1.28	0.211
Time (months)	11.63 ± 7.11	17.81 ± 14.06	1.57	0.127
FM	46.13 ± 15.17	45.81 ± 15.18	0.058	0.954
MBI	87.19 ± 13.16	90.31 ± 16.07	0.602	0.552

Left: stroke subjects with the left hemispheric lesion; right: stroke subjects with the right hemispheric lesion; site: site of the lesion; BG: basal ganglia; BS: brain stem; M: male; F: female; R: right handed; L: left handed; time: time poststroke; FM: Fugl-Meyer scores (upper extremity part); MBI: modified Barthel Index.

**Table 2:** Repeated measures ANOVA results of channels with statistical differences.

	Channels	SS	df	MS	*F*	Sig.
Alpha1	F7	7.603	2.119	3.587	4.276	0.017^∗^
	F4	3.508	2.3	1.525	3.471	0.031^∗^
	F6	4.078	2.348	1.737	4.633	0.009^∗∗^
	F8	3.548	2.109	1.682	5.450	0.006^∗∗^
	FT7	6.938	2.057	3.373	4.184	0.019^∗^
	T7	3.467	3	1.156	3.225	0.026^∗^
	FT8	2.964	2.162	1.371	4.439	0.014^∗^
	T7	3.467	3	1.156	3.225	0.026^∗^
	CP6	4.082	1.992	2.049	3.783	0.029^∗^
Alpha2	AF4	3.291	2.370	1.388	4.017	0.017^∗^
	F2	1.961	3	0.654	3.756	0.014^∗^
	F4	2.334	3	0.778	3.949	0.011^∗^
	F6	1.881	3	0.627	3.504	0.019^∗^
	F8	2.918	1.928	1.513	4.659	0.014^∗^
	FT8	1.390	2.348	0.592	3.959	0.018^∗^
	PO3	10.445	1.562	6.687	3.573	0.047^∗^
	POZ	5.666	2.019	2.807	3.266	0.045^∗^
	O1	6.041	2.115	2.857	3.212	0.045^∗^

SS: sum of squares (type III); df: degree of freedom; MS: mean squares. ^∗^*p* < 0.05, ^∗∗^*p* < 0.01.

## Data Availability

The EEG data and clinical characteristics of stroke subjects are restricted by the Tianjin Union Medical Centre, in order to protect patients' privacy. Data is available from the corresponding author of the paper for researchers who meet the criteria for access to the confidential data.

## Supplementary Materials

Supplementary Materials Supplementary Table 1 shows the randomized stimulation sequence of four sessions of the experiment.
