# Supplementary material for: Varied Response of EEG Rhythm to Different tDCS Protocols and Lesion Hemispheres in Stroke Subjects with Upper Limb Dysfunction
Source: Neural Plast. 2022 Jul 30;2022:7790730. doi: 10.1155/2022/7790730 (PMC9356883; doi:10.1155/2022/7790730)
Supplement: Supplementary Materials — Supplementary Table 1 shows the randomized stimulation sequence of four sessions of the experiment. [file 7790730.f1.docx]

Supplemental Table 1 Randomized stimulation sequence of four sessions of the experiment

| Subject | Session 1 | Session 2 | Session 3 | Session 4 |
| --- | --- | --- | --- | --- |
| 1 | 1 | 3 | 2 | 4 |
| 2 | 4 | 3 | 1 | 2 |
| 3 | 4 | 3 | 2 | 1 |
| 4 | 3 | 4 | 2 | 1 |
| 5 | 2 | 4 | 3 | 1 |
| 6 | 4 | 1 | 2 | 3 |
| 7 | 4 | 3 | 1 | 2 |
| 8 | 4 | 1 | 3 | 2 |
| 9 | 3 | 2 | 4 | 1 |
| 10 | 1 | 2 | 3 | 4 |
| 11 | 2 | 3 | 4 | 1 |
| 12 | 2 | 3 | 4 | 1 |
| 13 | 4 | 3 | 1 | 2 |
| 14 | 4 | 2 | 1 | 3 |
| 15 | 2 | 3 | 4 | 1 |
| 16 | 2 | 4 | 1 | 3 |
| 17 | 4 | 2 | 3 | 1 |
| 18 | 2 | 4 | 1 | 3 |
| 19 | 1 | 4 | 3 | 2 |
| 20 | 1 | 2 | 3 | 4 |
| 21 | 4 | 2 | 3 | 1 |
| 22 | 4 | 3 | 2 | 1 |
| 23 | 2 | 4 | 3 | 1 |
| 24 | 4 | 1 | 2 | 3 |
| 25 | 4 | 1 | 3 | 2 |
| 26 | 2 | 4 | 1 | 3 |
| 27 | 1 | 2 | 3 | 4 |
| 28 | 2 | 4 | 3 | 1 |
| 29 | 2 | 3 | 4 | 1 |
| 30 | 4 | 1 | 3 | 2 |
| 31 | 2 | 3 | 4 | 1 |
| 32 | 2 | 4 | 3 | 1 |

Note: 1 represents the anodal transcranial direct current stimulation; 2 represents the cathodal transcranial direct current stimulation; 3 represents the bilateral transcranial direct current stimulation; 4 represents the sham stimulation. The red”1”represents the missing session of subject 31.
